# Do Feed Plants Provide Sufficient Sodium, Calcium and Magnesium to Sika Deer in Japan? An Analysis Using Global Plant Trait Data

**DOI:** 10.3390/ani13061044

**Published:** 2023-03-13

**Authors:** Taiki Mori, Sho Iwagami, Hiromi Yamagawa, Kei K. Suzuki

**Affiliations:** 1 Kyushu Research Center, Forestry and Forest Products Research Institute, Kurokami 4-11-16, Kumamoto 860-0862, Japan; 2 Department of Disaster Prevention, Meteorology and Hydrology, Forestry and Forest Products Research Institute, FFPRI, Matsunosato 1, Tsukuba 305-8687, Japan

**Keywords:** *Cervus nippon*, mineral requirements, sodium, TRY plant traits database

## Abstract

**Simple Summary:**

Selective culling of female deer is effective in reducing overabundant sika deer (*Cervus nippon*) populations. Sodium (Na), calcium (Ca) and magnesium (Mg) can act as an attractant to selectively cull female sika deer due to the differences in mineral requirements. Here, using global plant trait data and a published list of sika deer feed plants in Japan, we estimated whether feed plants provide sika deer sufficient Na, Ca and Mg, and compared the results between males and females. Our analysis showed that sufficient Na and Ca were not always provided, especially when intakes were low and deer were large. Na deficiency was more intense for lactating females than males, whereas Ca deficiency was more intense for males. We suggest that Na and Ca could be useful for developing effective culling methods. Especially, Na could be useful for selective culling of female sika deer during the lactating period.

**Abstract:**

Deficient minerals in overabundant populations could act as an attractant to cull sika deer (*Cervus nippon*). Because selective culling of female deer is reported to be effective in reducing sika deer populations, it is particularly important to clarify the differences in mineral requirements between males and females. Here, using global plant trait data and a published list of sika deer feed plants in Japan, we estimated whether feed plants provide sika deer sufficient sodium (Na), calcium (Ca) and magnesium (Mg), and compared the results between males and females. An analysis of 191 feed plant species suggested that feed plants can provide sufficient Mg, whereas sufficient Na and Ca is not always provided, especially when the intake is small or the deer large. Na deficiency was more intense for lactating females than males, suggesting that Na can be an effective attractant for selectively culling female deer. In summary, this study demonstrated that sika deer in Japan might require extra Na and Ca sources in addition to feed plants, and therefore these minerals could be useful for developing effective culling methods.

## 1. Introduction

Ungulate overpopulation is an important issue in the northern hemisphere [1], including northern America [2] and Europe [3]. Ungulate populations are regulated by bottom-up, top-down and abiotic factors [4]. A lack of certain nutrients/elements may limit ungulate population growth, especially at a high population density.

Sodium (Na), calcium (Ca) and magnesium (Mg) may be limiting elements for ungulates at a high population density because livestock are often deficient in these elements, especially Na, which is supplemented artificially [5]. A mineral deficiency can also occur in nature based on the fact that various animals use salt licks [6,7,8]. Geophagy (i.e., eating soil) is common in ungulates [9,10,11], probably for mineral supplementation. Various species were reported to lick natural springs, presumably for obtaining minerals [12]. Ceacero et al. (2014) [13] reported that Iberian red deer (*Cervus elaphus*) feed on seaweeds, possibly to acquire minerals (but see Ceacero et al., 2015 [14]). The use of salt licks has often been attributed to a requirement for Na [8,9,15], which is important for maintaining osmotic pressure [16]. However, it may also be due to a requirement for other minerals, such as Ca, which is a major component of bones [17], and Mg, which has important roles in enzyme activity and bone development [18]. Several studies have argued that natural salt licks are important sources of Ca and Mg [19].

Sika deer (*Cervus nippon*) are native to eastern Asia and have been introduced into many parts of the world, such as Europe, North America and New Zealand [20]. In some areas, the deer populations have increased enough that they cause serious browsing damage to natural and anthropogenic environments [20,21]. In Japan, especially, the sika deer population has increased dramatically in recent decades [22]. According to the Ministry of the Environment (2015), the distribution of sika deer expanded more than 2.5 times from 1978 to 2015 [23]. Accordingly, sika deer are being culled in many parts of Japan to reduce overabundant populations, and the development of effective culling methods is desirable. Since this substantial increase in the sika deer population and distribution and subsequent overbrowsing could lead to mineral deficiencies in sika deer, we considered that the deficient minerals in overabundant populations act as an attractant to cull deer. Indeed, sika deer also lick salt [24]. Furthermore, it is particularly important to clarify the differences in mineral deficiency between males and females because selective culling of female deer is reported to be effective in reducing sika deer populations [25,26]. However, no study has evaluated whether sika deer in Japan obtain sufficient minerals, such as Na, Ca and Mg, through feed plants by comparing the minerals provided by feed plants and the mineral requirements of sika deer. Mineral deficiencies between male and female deer have also not been compared.

In this study, we evaluated whether feed plants can provide sika deer sufficient Na, Ca and Mg by comparing the minerals provided by feed plants, calculated from leaf mineral contents and mineral requirements of sika deer, simulated by several scenarios of daily dry matter intake (DMI), body weight (BW) and sex. Since the feed plant species composition of wild sika deer in their natural state is currently undeterminable, we investigated whether each plant species could provide sufficient minerals to sika deer when sika deer ingested only that plant species. It should be noted that this approach may be misleading because this approach did not consider the feed plant species composition of wild sika deer and therefore is unable to accurately estimate the amount of minerals provided by feed plants. However, this approach is valuable to roughly estimate the amount of mineral deficiency in wild sika deer under natural conditions, which is the first attempt in Japan.

## 2. Materials and Methods

### 2.1. Dataset

A dataset of leaf nutrient contents of sika deer feed plants in Japan was constructed using plant trait data from the TRY Plant Trait Database [27] and a list of feed plant species of sika deer in Japan [28]. From TRY, we extracted the leaf Na, Ca and Mg contents per leaf dry mass (Trait IDs 260, 252 and 257, respectively) for plant species previously documented as sika deer feed in Japan [28]. In other words, plants not recorded as feed plants, including toxic plants [14], were not included in the analysis. The final dataset included 3179 values for 191 plant species (Table S1) from five original publications [29,30,31,32,33]. The leaf mineral content data were averaged for each species, and the averaged values were used for further analysis.

### 2.2. Analysis

Using the leaf mineral concentration data for sika deer feed plants in Japan, we simulated whether each plant species could provide sufficient minerals for sika deer. We considered four sika deer body weight (BW) scenarios based on the BW range of sika deer in Japan (i.e., 30, 60, 90 and 120 kg) and four daily dry matter intake (DMI) scenarios considering a wider range than reported from feeding experiments (i.e., 0.5, 1.5, 3 and 4.5 kg) [34,35,36]. Unrealistic scenarios (BW 30 kg with DMI 3 or 4.5 kg; BW 60 kg with DMI 4.5 kg; and BW 120 kg with DMI 0.5 kg) were excluded from our analysis. Mineral intakes provided by leaves, which were calculated by multiplying DMI and leaf mineral concentration, were compared with the mineral requirement of sika deer calculated as follows. The Na maintenance requirement was calculated based on the following equation for *Cervus* [17]:

*Na_req_maintain* = 9.0/1000 × *BW* (g day^−1^) (1)

where *Na_req_maintain* is the Na requirement for maintenance and *BW* is body weight (kg). The Na requirement for males was calculated as *Na_req_maintain* plus the requirement for antlers, assuming that antler growth in sika deer requires 150 days, as follows [17]:

*Na_req_male* = *Na_req_maintain* + (0.005 × 26 × *BW*^0.75^)/(0.98 × 150) (g day^−1^) (2)

The equation for determining the Na requirement of females during lactation [17] was modified for sika deer as follows:

*Na_req_female* = *Na_req_maintain* + (0.0175 × *BW* × *Na_milk_sika*)/0.98 (3)

where *Na_milk_sika* is the Na content in sika deer milk (1.00 g L^−1^, [37]). We also calculated the Na requirement of females during the non-lactating period (equal to *Na_req_maintain*) by omitting the term expressing the Na requirement for milk production from the equation. The Na requirement of females during the gestation period is shown in Figure S1. The above equations do not include the Na requirements for body growth, assuming that body growth is limited by a lack of feed plants. This is because we set conservative thresholds to avoid overestimating the mineral deficiency. The maintenance Mg requirement (*Mg_req_maintain*) was calculated based on the following equation for goats [17], because equations for goats were recommended to calculate values for cervids (equations for cervids were not available) [17]:

*Mg_req_maintain* = (0.0035 × *BW*)/0.20 (g day^−1^) (4)

Without considering Mg requirement for body growth, the Mg requirement for males was assumed to be equal to *Mg_req_maintain* [17]. The Mg requirement for lactating females was calculated using equations prepared for goats as recommended [17], but modified as follows:

*Mg_req_female* = *Mg_req_maintain* + (0.0175 × *BW* × *Mg_milk_sika*)/0.20 (5)

where *Mg_milk_sika* is the Mg content of sika deer milk (0.0819 g L^−1^ [37]). The Mg requirement of females during the non-lactating period was considered equal to the Mg requirement of males. The Mg requirement of females during the gestation period was shown in Figure S2. The maintenance Ca requirement (*Ca_req_maintain*) was calculated based on the following equation for *Cervus* [17]:

*Ca_req_maintain* = (0.025 × *BW* + 0.25 × *DMI*)/0.34 (g day^−1^) (6)

The Ca requirement for males was calculated as *Ca_req_maintain* plus the Ca requirement for antlers as [17]:

*Ca_req_male* = *Ca_req_maintain* + (0.05 × *BW*)/0.39 (g day^−1^) (7)

The Ca requirement for lactating females was calculated as *Ca_req_maintain* plus the Ca requirement for lactation [17]. We modified the equation by replacing the milk Ca concentration of the *Cervus* with that of sika deer [37]:

*Ca_req_female* = *Ca_req_maintain* + (0.0175 × *BW* × *Ca_milk_sika*)/0.39 (8)

where *Ca_milk_sika* is the Ca content of sika deer milk (1.62 g L^−1^ [37]). The Ca requirements for body growth were also not included in the same manner as Na and Mg. The Ca requirement of females during the non-lactating period (equal to *Ca_req_maintain*) was also calculated by omitting the term expressing the Ca requirement for milk production from the equation. The Ca requirement of females during the gestation period was shown in Figure S3.

## 3. Results

**Figure 1:**
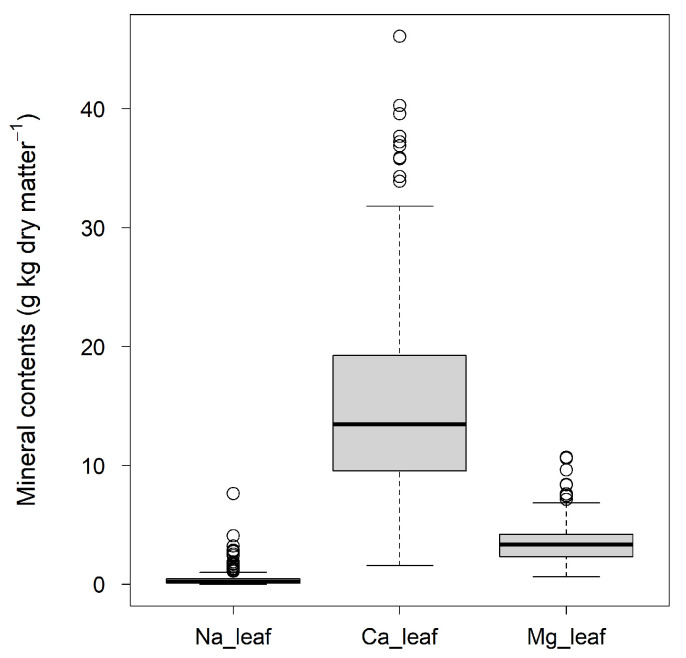
Boxplot of the distribution of leaf mineral contents of sika deer feed plants. Na_leaf, Ca_leaf and Mg_leaf are the Na, Ca and Mg contents per leaf dry mass, respectively.

The sika deer feed plant dataset used for this analysis showed large variance (Figure 1). The average leaf contents of Na, Ca and Mg were 0.47 ± 0.82, 15.3 ± 8.4 and 3.5 ± 1.8 (g element kg dry matter^−1^ ± standard deviation), respectively.

**Figure 2:**
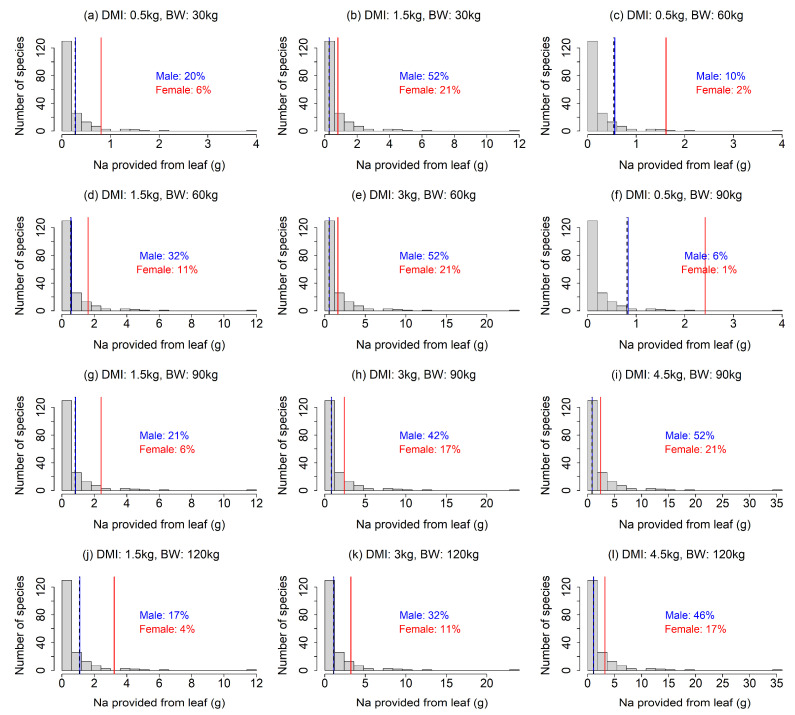
(**a**–**l**) Comparison of Na provision by feed plants and the Na requirement of sika deer under 12 scenarios (four dry matter intake levels × four body weights, excluding unrealistic scenarios). The histogram indicates the distribution of Na provision calculated from the leaves of feed plant species. The solid lines indicate the Na requirements of male (blue) and female (red) sika deer. Dashed line indicates Na requirement of females during the non-lactating period, which is equal to Na maintenance requirement for both males and females. In the figures, the percentage is the proportion of feed plant species that meet the Na requirements.

**Figure 3:**
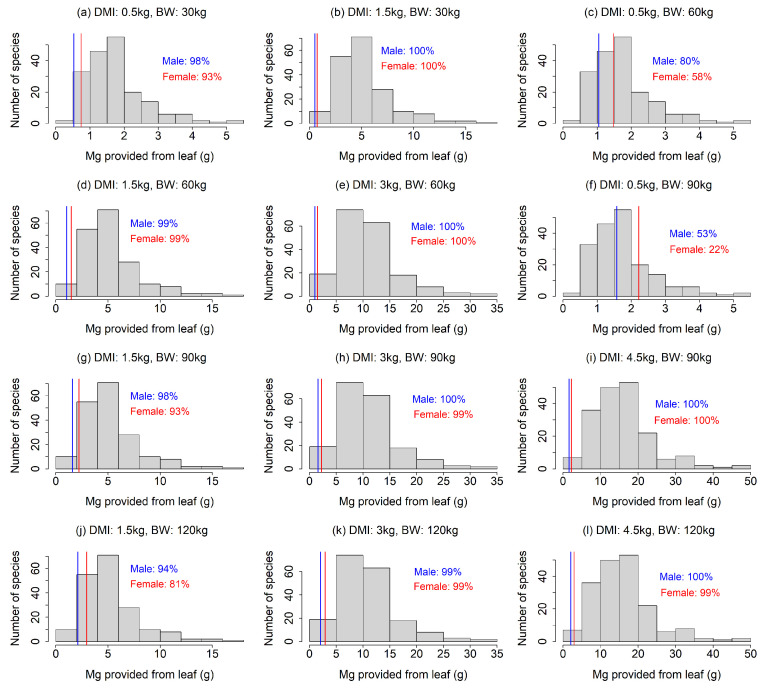
(**a**–**l**) Comparison of Mg provision by feed plants and the Mg requirement of sika deer under 12 scenarios (four dry matter intake levels × four body weights, excluding unrealistic scenarios). The histogram indicates the distribution of Mg provision calculated from the leaves of feed plant species. The solid lines indicate the Mg requirements of male (blue) and female (red) sika deer. Mg requirement of females during the non-lactating period is equal to the Mg requirement of males. In the figures, the percentage is the proportion of feed plant species that meet the Mg requirements.

**Figure 4:**
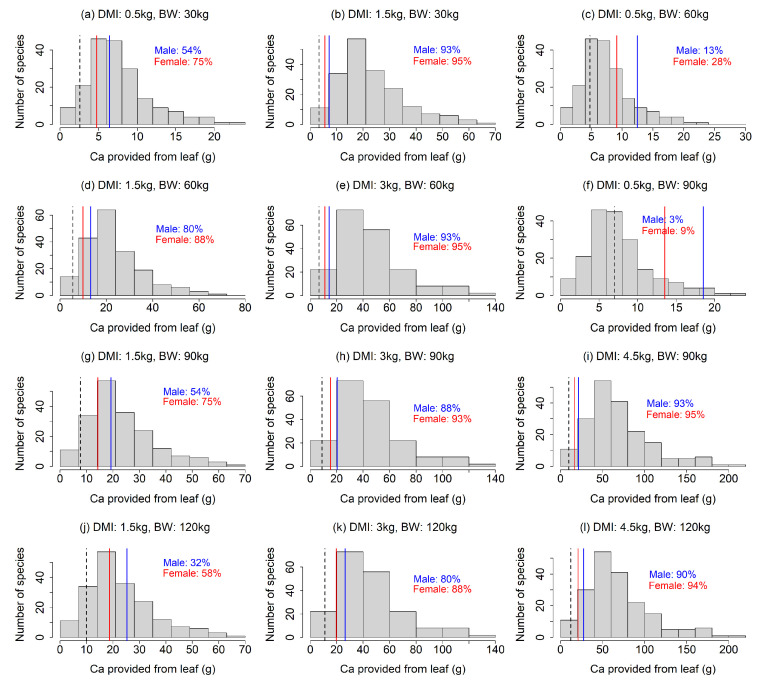
(**a**–**l**) Comparison between Ca provision via feed plants and the Ca requirement of sika deer under the 12 scenarios (four dry matter intake levels × four body weights, excluding unrealistic scenarios). The histogram is the distribution of Ca provision calculated from leaves of feed plant species. The solid lines indicate the Ca requirements of male (blue) and female (red) sika deer. Dashed line indicates the Ca requirement of females during the non-lactating period, which is equal to the Na maintenance requirement for both males and females. In the figures, the percentage is the proportion of feed plant species that met the Ca requirements.

Our analysis showed that the Na requirements were rarely achieved by feed plants, especially under the scenarios with a smaller DMI and larger BW (Figure 2). In most of the 12 scenarios, less than 50% of the feed plant species provided sufficient Na (Figure 2a,c,d,f–h,j–l). The results also indicated that a Na deficiency would be more intense for lactating females than males (Figure 2), as the Na requirement for females was more than double that of males (Figure 2). Our finding that Na requirements often exceed the provision by feed plants contrasted to that for Mg; the leaf Mg contents were higher than required in most of the feed plants in our analysis, except for the scenarios with combinations of large BW and small DMI (Figure 3c,f), indicating that Mg deficiency is less likely. The fulfillment of the Ca requirement depended largely on the scenario (Figure 4). A lower DMI resulted in insufficient Ca provision in a large portion of the feed plant species, especially for greater BWs (Figure 4a,c,f,g,j), whereas Ca requirements were met in a large portion of the feed plant species when the DMI was ≥3 kg (Figure 4). The Ca requirement for males was larger than that of females (Figure 4). 

## 4. Discussion

Our results suggest that feed plants in Japan are unlikely to provide sufficient Na, which agrees with the traditional view that Na is the main deficient mineral leading to the use of salt licks [8,9,15]. By comparing several salt solutions as attractants experimentally, Fraser and Reardon (1980) demonstrated that Na, but not K, Ca or Mg, attracted moose (*Alces alces*) and white-tailed deer (*Odocoileus virginianus*), indicating that Na was the primary attractant at their study site [6]. Sika deer were also reported to be attracted by Na [15,24,38]. The larger Na deficiency for lactating females than males (Figure 2) is consistent with reports that female sika deer were attracted by salt, especially during pregnancy or lactation [15,24].

This study suggests the importance of Ca as a potentially limiting element of sika deer in Japan. Although natural salt licks have been attributed to Na acquisition, several authors have reported that Ca could also be a salt lick attractant [19]. Kitahara et al. (2005) reported that sika deer in Hokkaido, Japan may be Ca-deficient [39]. Tsujii and Tokumoto (1997) reported that several natural salt licks in Japan did not have high Na contents [40], but they tended to have high Ca, Fe, Mg and Mn contents. Miyazaki and Kohara (2017) reported that sika deer licked an antifreeze containing calcium chloride (CaCl_2_) as the main component, proposing that antifreezes accelerate the overpopulation of sika deer in Japan by providing Ca [41]. Our simulation supported the hypothesis that sika deer in Japan are potentially deficient in Ca, demonstrating that Ca requirements may not be met by feed plants, at least in certain situations (Figure 4). This study also raised the question of how sika deer acquire minerals in high-density areas with small amounts of feed plants, if they do not have access to salt licking sites.

Our finding might be beneficial for efficiently culling sika deer using minerals as attractants. Culling is one of the important management tools for overabundant sika deer populations [25,42], and suitable attractants have been verified to increase culling efficiency [43,44]. We suggest that Na can be an effective attractant for selectively culling female deer, which has been reported to be effective in reducing sika deer populations [25,26], especially during the lactating period (Figure 2). We also suggest that Ca could be a potential attractant for sika deer (Figure 4), as well as Na, which has been used as an effective attractant for hunting sika deer [24]. However, since Ca deficiency is stronger in male deer, Ca may not be an effective attractant for selective culling of females. Given the different mineral requirement patterns observed for males and females (Figure 2 and Figure 4) and potential seasonal changes in the effects of minerals to attract males and females [15], the combination of multiple mineral usage depending on seasons might be more effective for sika deer culling.

In summary, our results indicate that feed plants are unlikely to always provide sufficient Na and Ca, especially under current conditions in Japan, where sika deer overpopulation has intensified herbivorous pressure and the choice of feed plant species is often limited. Furthermore, we suggested that Na can be an effective attractant for selectively culling female deer, whereas Ca could be an attractant for male deer. Nevertheless, this study has several limitations. First, the data were obtained from a global database, and thus the analysis might not be applicable to a specific region. In the future study, data should be collected on a local scale, as plant mineral content is affected by soil conditions and climate. Second, this approach did not consider the feed plant species composition of wild sika deer and therefore is unable to accurately estimate the amount of minerals provided by feed plants. Quantitative data on the feed plant species composition of wild sika deer is necessary to more accurately estimate the mineral supply to sika deer. Third, the equations used to calculate the Na, Ca and Mg requirements were not constructed for sika deer, although a minor modification was made for sika deer (see Section 2 Materials and Methods). Equations for sika deer are required for a more robust estimation. Despite these limitations, this study demonstrates that sika deer in Japan might require extra Na and Ca sources in addition to feed plants, and therefore these minerals could be useful for developing effective culling methods.

## Data Availability

Not applicable.

## Supplementary Materials

The following supporting information can be downloaded at: https://www.mdpi.com/article/10.3390/ani13061044/s1, Figure S1: Comparison between Na provision via feed plants and the Na requirement of sika deer under the 12 scenarios; Figure S2: Comparison between Mg provision via feed plants and the Mg requirement of sika deer under the 12 scenarios; Figure S3: Comparison between Ca provision via feed plants and the Ca requirement of sika deer under the 12 scenarios; Table S1: Feed plant species included in the present study.

## References

[1] Côté S.D., Rooney T.P., Tremblay J.P., Dussault C., Waller D.M. (2004). Ecological impacts of deer overabundance. Annu. Rev. Ecol. Evol. Syst..

[2] Mcshea W.J. (2012). Ecology and management of white-tailed deer in a changing world. Ann. N. Y. Acad. Sci..

[3] Carpio A.J., Apollonio M., Acevedo P. (2021). Wild ungulate overabundance in Europe: Contexts, causes, monitoring and management recommendations. Mamm. Rev..

[4] Vucetich J.A., Peterson R.O. (2004). The influence of top-down, bottom-up and abiotic factors on the moose (*Alces alces*) population of Isle Royale. Proc. R. Soc. B Biol. Sci..

[5] Rauch R.E., Robinson P.H., Erasmus L.J. (2012). Effects of sodium bicarbonate and calcium magnesium carbonate supplementation on performance of high producing dairy cows. Anim. Feed Sci. Technol..

[6] Fraser A.D., Reardon E. (1980). Attraction of Wild Ungulates to Mineral-Rich Springs in Central Canada. Ecography.

[7] Panichev A.M., Zaumyslova O.Y.U., Aramilev V.V. (2002). The importance of salt licks and other sources of sodium in the ecology of the Ussuri moose (*Alces alces* cameloides). Alces J. Devoted Biol. Manag. Moose.

[8] Matsubayashi H., Lagan P., Majalap N., Tangah J., Sukor J.R.A., Kitayama K. (2007). Importance of natural licks for the mammals in Bornean inland tropical rain forests. Ecol. Res..

[9] Tracy B.F., McNaughton S.J. (1995). Elemental analysis of mineral lick soils from the Serengeti National Park, the Konza Prairie and Yellowstone National Park. Ecography.

[10] Klaus G., Klaus-Hügi C., Schmid B. (1998). Geophagy by large mammals at natural licks in the rain forest of the Dzanga National Park, Central African Republic. J. Trop. Ecol..

[11] Ayotte J.B., Parker K.L., Arocena J.M., Gillingham M.P. (2006). Chemical composition of lick soils: Functions of soil ingestion by four ungulate species. J. Mammal..

[12] Fraser D., Hristienko H. (2000). Activity of Moose and White-Tailed Deer at Mineral Springs. Can. J. Zool..

[13] Ceacero F., Landete-Castillejos T., Miranda M., García A.J., Martínez A., Gallego L. (2014). Why do cervids feed on aquatic vegetation?. Behav. Processes.

[14] Ceacero F., Landete-Castillejos T., Olguín A., Miranda M., García A., Martínez A., Cassinello J., Miguel V., Gallego L. (2015). Avoiding toxic levels of essential minerals: A forgotten factor in deer diet preferences. PLoS ONE.

[15] Ping X., Li C., Jiang Z., Liu W., Zhu H. (2011). Sexual difference in seasonal patterns of salt lick use by south China sika deer Cervus nippon. Mamm. Biol..

[16] Suttle N.F. (2010). Mineral Nutrition of Livestock.

[17] NRC N.R.C. (2007). Nutrient Requirements of Small Ruminants.

[18] Pinotti L., Manoni M., Ferrari L., Tretola M., Cazzola R., Givens I. (2021). Human Nutrition. Nutrients.

[19] Jones R.L., Hanson H.C. (1985). Mineral Licks, Geophagy, and Biogeochemistry of North American Ungulates.

[20] McCullough D., Takatsuki S., Kaji K. (2009). Sika Deer: Biology and Management of Native and Introduced Populations.

[21] Putman R.J., Moore N.P. (1998). Impact of deer in lowland Britain on agriculture, forestry and conservation habitats. Mamm. Rev..

[22] Takatsuki S. (2009). Effects of sika deer on vegetation in Japan: A review. Biol. Conserv..

[23] Ministry of the Environment (2015). Estimation of Sika Deer Population in Japan. http://www.env.go.jp/press/100922.html.

[24] Yasuda M., Suzuki K.K. (2022). Dear and minerals. J. Jpn. Wildl. Res. Soc..

[25] Ueno M., Kaji K., Saitoh T. (2010). Culling Versus Density Effects in Management of a Deer Population. J. Wildl. Manag..

[26] Suzuki K., Kuwano Y., Yasuda M. (2022). A 17 year study of the response of populations to different patterns in antlerless proportion of imposed culls: Antlerless culling reduces overabundant deer population. Biology.

[27] Kattge J., Bönisch G., Díaz S., Lavorel S., Prentice I.C., Leadley P., Tautenhahn S., Werner G.D.A., Aakala T., Abedi M. (2020). TRY plant trait database—Enhanced coverage and open access. Glob. Change Biol..

[28] Hashimoto Y., Fujiki D. (2014). List of food plants and unpalatable plants of sika deer (*Cervus nippon*) in Japan Yoshinobu. Hum. Nat..

[29] Nakanishi T., Atarashi-Andoh M., Koarashi J., Saito-Kokubu Y., Hirai K. (2012). Carbon isotopes of water-extractable organic carbon in a depth profile of forest soil imply a dynamic relationship with soil carbon. Eur. J. Soil Sci..

[30] Watanabe T., Broadley M.R., Jansen S., White P.J., Takada J., Satake K., Takamatsu T., Tuah S.J., Osaki M. (2007). Evolutionary control of leaf element composition in plants: Rapid report. New Phytol..

[31] Penuelas J., Sardans J., Llusià J., Owen S.M., Carnicer J., Giambelluca T.W., Rezende E.L., Waite M., Niinemets Ü. (2010). Faster returns on “leaf economics” and different biogeochemical niche in invasive compared with native plant species. Glob. Change Biol..

[32] Herz K., Dietz S., Haider S., Jandt U., Scheel D., Bruelheide H. (2017). Drivers of intraspecific trait variation of grass and forb species in German meadows and pastures. J. Veg. Sci..

[33] Schmitt M., Mehltreter K., Sundue M., Testo W., Watanabe T., Jansen S. (2017). The evolution of aluminum accumulation in ferns and lycophytes. Am. J. Bot..

[34] Souma K., Masuko T., Kobayashi Y., Ishijima Y. (1998). Seasonal alteration of hay intake in the Yeso sika deer (*Cervus nippon* yesoensis). Hokushinetsu J. Anim. Sci..

[35] Yamane M. (2015). Over-winter foraging activities of supplementally fed free-ranging Sika deer (*Cervus nippon*). Bull. Kanagawa Prefect. Nat. Environ. Conserv. Cent..

[36] Tamura T., Terasaki T., Oikawa M., Kaji K., Nara M., Arai K., Nakamura K. (2012). The effect of seasonal transformation on feed intake in Sika deer Tetsuo. Bull. Tokyo Metrop. Agric. For. Res. Cent..

[37] Ishida M., Meguro J., Ikeda S., Takeda T. (1995). Mineral analysis of meat, milk and velvet of Japanese deer. Sci. Rep. Miyagi Agric. Coll..

[38] Yasuda M., Yayota C. (2020). A trial creating artificial salt lick in forest. Kyushu J. For. Res..

[39] Kitahara R., Komatsu T., Masuko T. (2005). Effects of mineral requirements on food selection by sika deer (*Cervus nippon* yesoensis). Rep. Pro Nat. Fund.

[40] Tsujii H., Tokumoto T. (1997). Soil Licking Phenomenon of Wild Sika Deer (*Cervus nippon*). Around the origin of Gohukuji river at Matsumoto City. Hokuriku J. Zootech. Sci..

[41] Miyazaki M., Kohara M. (2017). Mori No Tantei.

[42] Suzuki K.K., Yasuda M., Sonoda M. (2022). Spatially biased reduction of browsing damage by sika deer through culling. J. Wildl. Manag..

[43] Iijima H., Oochi J. (2016). Examination of useful bait to attract sika deer. Mamm. Sci..

[44] Ikeda T., Shirakawa T., Suzuki M. (2018). Comparison of the attractiveness of five baits in sika deer (*Cervus nippon*) and wild boar (*Sus scrofa*). Wildl. Hum. Soc..

