# Supplementary material for: Do Feed Plants Provide Sufficient Sodium, Calcium and Magnesium to Sika Deer in Japan? An Analysis Using Global Plant Trait Data
Source: Animals (Basel). 2023 Mar 13;13(6):1044. doi: 10.3390/ani13061044 (PMC10044698; doi:10.3390/ani13061044)
Supplement: Supplementary file 1 [file animals-13-01044-s001.zip › animals-2179075-supplementary.pdf]

Supplemental Materials

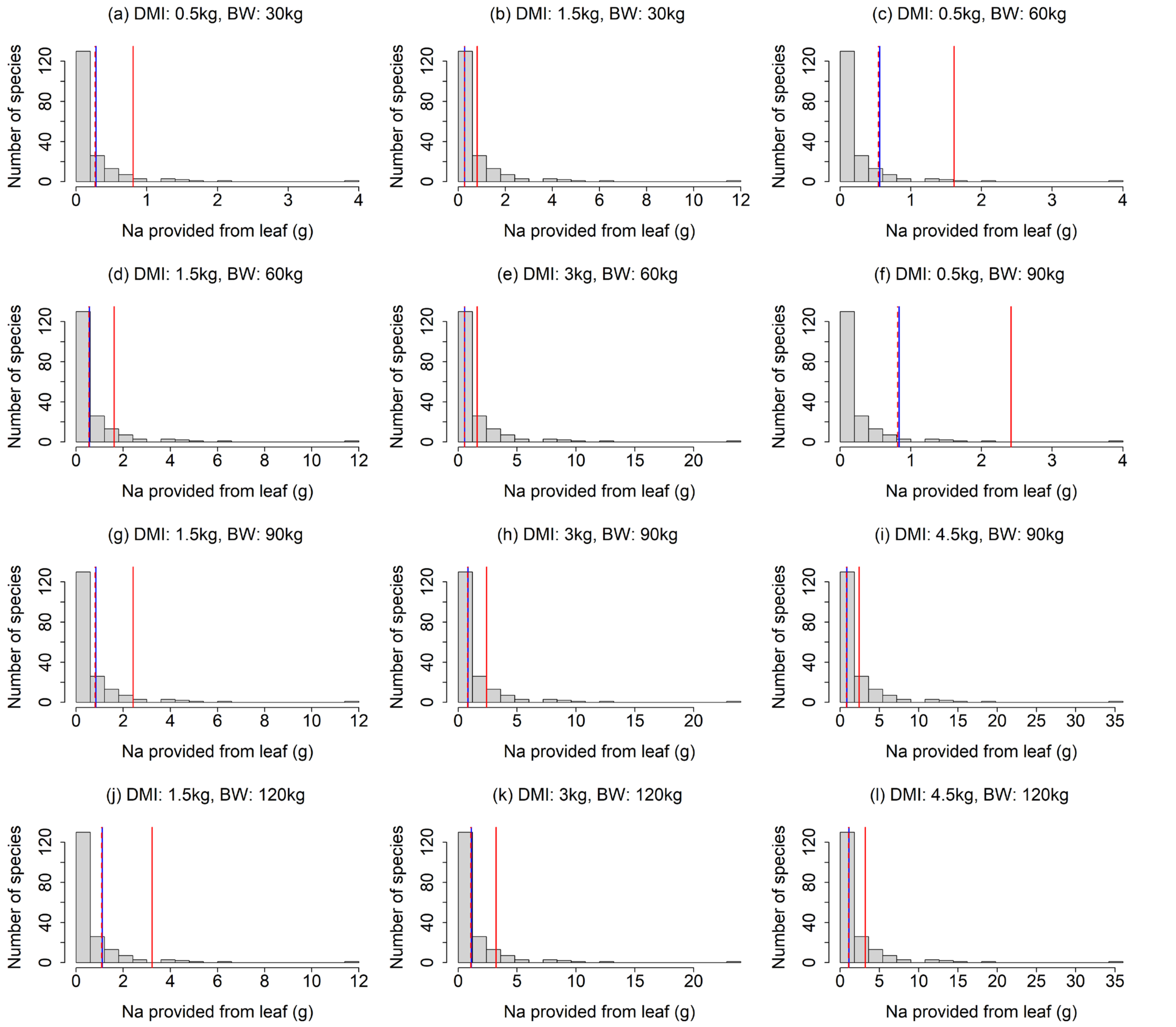

Figure S1. Comparison of Na provision by feed plants and the Na requirement of sika deer under 12 scenarios (four dry matter intake levels × four body weights excluding unrealistic scenarios). The histogram indicates the distribution of Na provision calculated from the leaves of feed plant species. The solid lines indicate the Na requirements of male (blue) and female (red) sika deer. Dashed red line indicates Na requirement of female during gestation period, which was calculated as following equation [17]. Na requirements = *Na_req_maintain* (eq. 1) + ($0.013 \times 0.166 \times BW^{0.9} \times 0.021$) / 0.98 (g day^{-1})

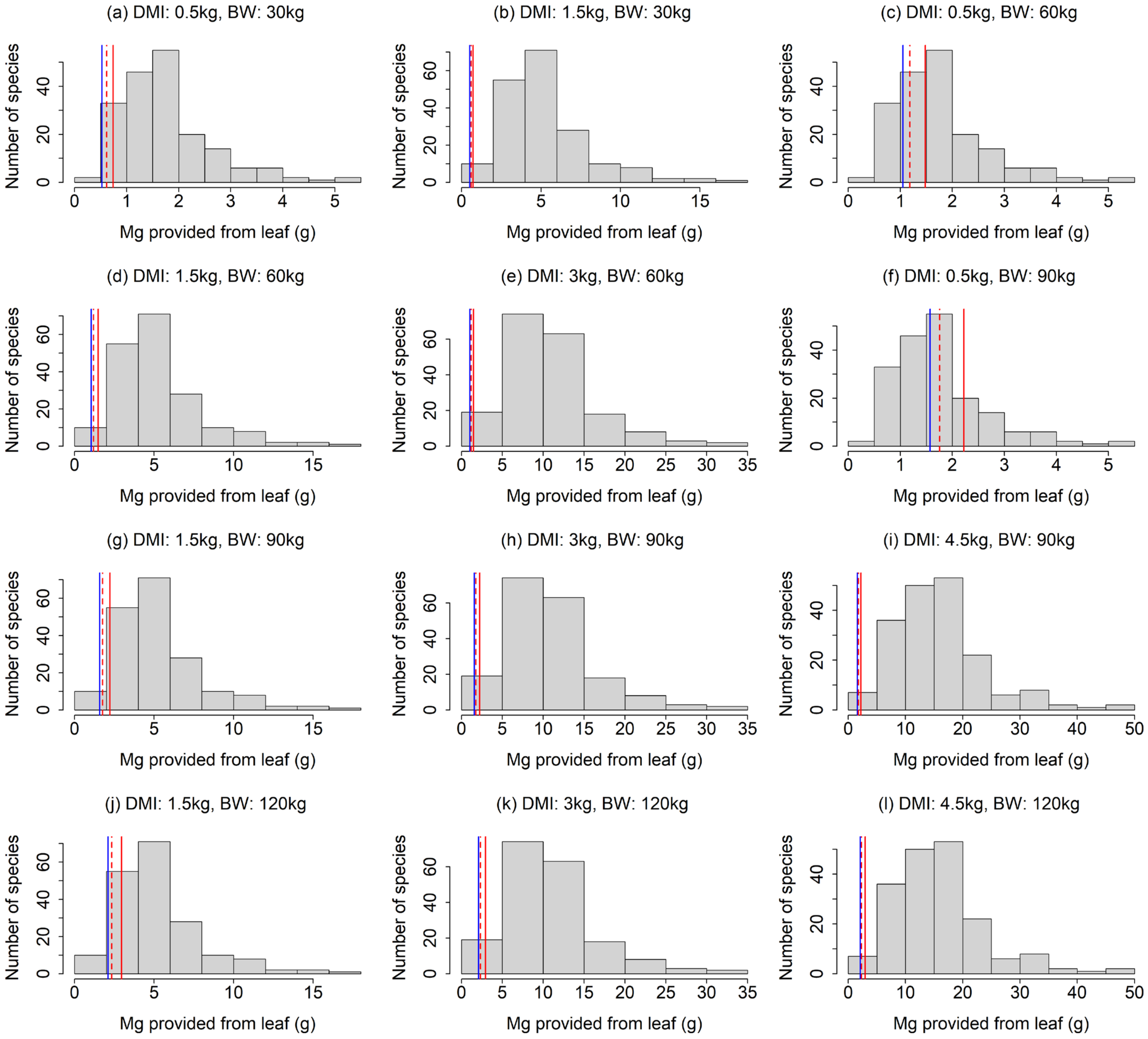

Figure S2. Comparison of Mg provision by feed plants and the Mg requirement of sika deer under 12 scenarios (four dry matter intake levels × four body weights excluding unrealistic scenarios). The histogram indicates the distribution of Mg provision calculated from the leaves of feed plant species. The solid lines indicate the Mg requirements of male (blue) and female (red) sika deer. Dashed red line indicates Mg requirement of female during gestation period, which was calculated as following equation [17]. Mg requirements = *Mg_req_maintain* (eq. 4) + (0.006 × *LBW*) / 0.2, where *LBW* indicates litter birth weight; *LBW* of 3.0, 4.5, 6.0 and 7.5 kg were used for *BW* 30, 60, 90 and 120 kg, respectively.

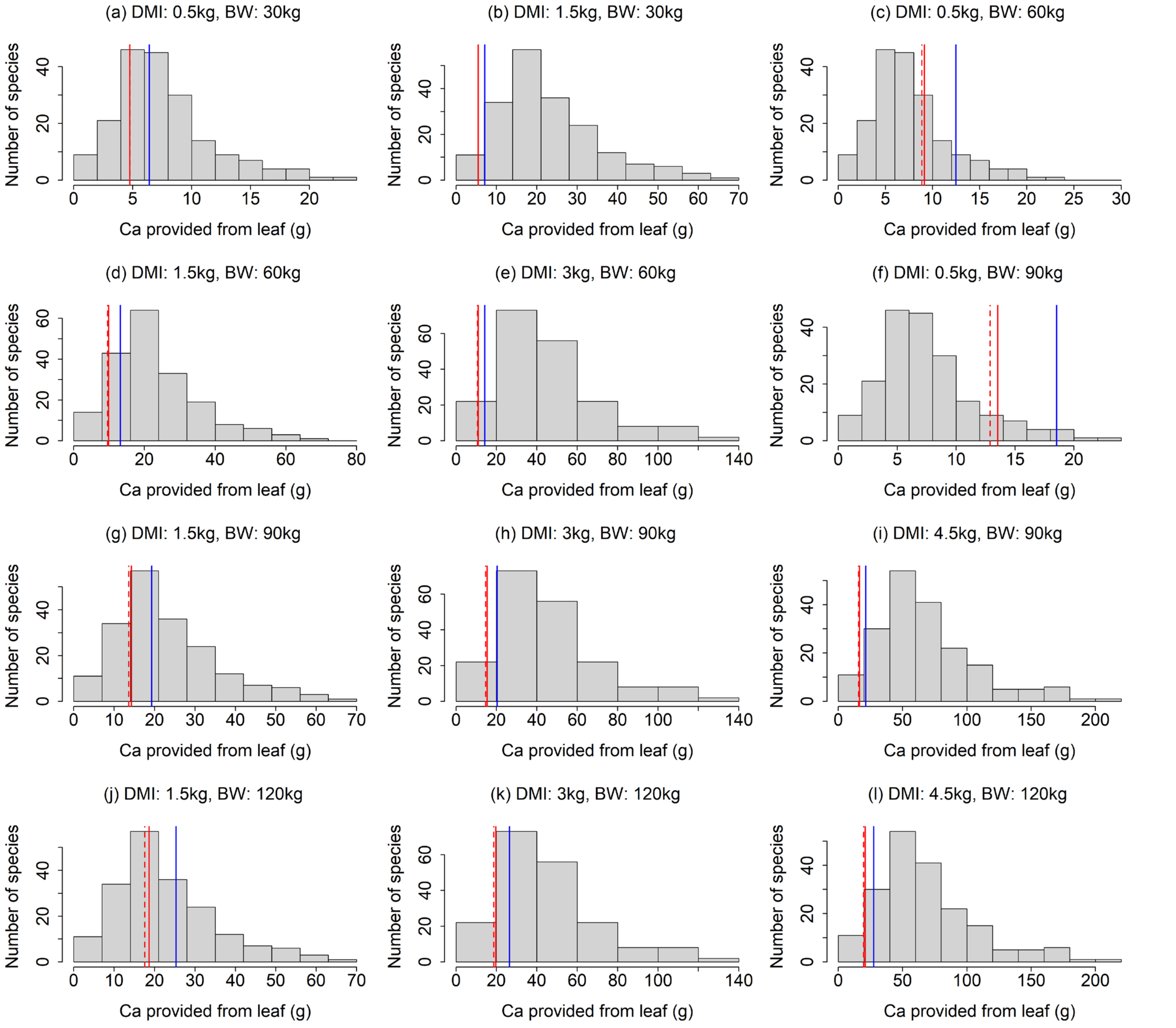

Figure S3. Comparison between Ca provision via feed plants and the Ca requirement of sika deer under the 12 scenarios (four dry matter intake levels × four body weights excluding unrealistic scenarios). The histogram is the distribution of Ca provision calculated from leaves of feed plant species. The solid lines indicate the Ca requirements of male (blue) and female (red) sika deer. Dashed red line indicates Ca requirement of female during gestation period, which was calculated as following equation [17]**.** Ca requirements = *Ca_req_maintain* (eq. 6) + $(11.5 \times 0.166 \times BW^{0.9} \times 0.021) / 0.39$ (g day^{-1})

Table S1. Feed plant species included in the present study.

No	Species	No	Species	No	Species
1	Abies sachalinensis	34	Camellia sinensis	67	Equisetum hyemale
2	Acacia confusa	35	Carpinus cordata	68	Euonymus alatus
3	Acer carpinifolium	36	Carpinus japonica	69	Euonymus japonicus
4	Acer japonicum	37	Carpinus laxiflora	70	Euonymus oxyphyllus
5	Acer palmatum	38	Carpinus tschonoskii	71	Euptelea polyandra
6	Acer pictum	39	Castanea crenata	72	Eurya emarginata
7	Acer rufinerve	40	Castanopsis cuspidata	73	Eurya japonica
8	Acer sieboldianum	41	Celtis sinensis	74	Euscaphis japonica
9	Actinidia arguta	42	Cephalotaxus harringtonia	75	Fagus crenata
10	Aesculus turbinata	43	Cercidiphyllum japonicum	76	Ficus erecta
11	Akebia quinata	44	Chamaecyparis obtusa	77	Firmiana simplex
12	Alangium platanifolium	45	Cinnamomum camphora	78	Fraxinus mandshurica
13	Albizia julibrissin	46	Cinnamomum tenuifolium	79	Fraxinus sieboldiana
14	Alnus firma	47	Cirsium japonicum	80	Gardenia jasminoides
15	Alnus hirsuta	48	Clerodendrum trichotomum	81	Ginkgo biloba
16	Alnus sieboldiana	49	Clethra barbinervis	82	Gleichenia japonica
17	Angelica pubescens	50	Cleyera japonica	83	Glochidion obovatum
18	Angiopteris lygodiifolia	51	Corylus sieboldiana	84	Glycine max
19	Antidesma japonicum	52	Cryptomeria japonica	85	Hamamelis japonica
20	Aphananthe aspera	53	Daphniphyllum macropodum	86	Helwingia japonica
21	Arachniodes aristata	54	Dendropanax trifidus	87	Huperzia serrata
22	Arachniodes standishii	55	Deutzia crenata	88	Hydrangea hirta
23	Aralia cordata	56	Dioscorea japonica	89	Hydrangea paniculata
24	Aralia elata	57	Diospyros kaki	90	Hydrangea petiolaris
25	Ardisia sieboldii	58	Diospyros lotus	91	Hypericum erectum
26	Aucuba japonica	59	Diospyros morrisiana	92	Ilex crenata
27	Betula grossa	60	Distylium racemosum	93	Ilex integra
28	Betula platyphylla	61	Dryopteris crassirhizoma	94	Ilex macropoda
29	Broussonetia kazinoki	62	Elaeagnus pungens	95	Ilex pedunculosa
30	Callicarpa dichotoma	63	Elaeagnus umbellata	96	Ilex serrata
31	Callicarpa japonica	64	Eleutherococcus senticosus	97	Illicium anisatum
32	Callicarpa mollis	65	Eleutherococcus spinosus	98	Larix kaempferi
33	Camellia japonica	66	Equisetum arvense	99	Lespedeza bicolor

100	Ligustrum japonicum	133	Pinus densiflora	166	Sorbus commixta
101	Ligustrum obtusifolium	134	Pinus parviflora	167	Spiraea salicifolia
102	Lindera erythrocarpa	135	Pinus thunbergii	168	Stachyurus praecox
103	Lindera obtusiloba	136	Plagiogyria matsumureana	169	Staphylea bumalda
104	Lindera umbellata	137	Poa pratensis	170	Stephanandra incisa
105	Lycopodium clavatum	138	Polystichum tripteron	171	Stewartia monadelpha
106	Lyonia ovalifolia	139	Populus tremula var. sieboldii	172	Stewartia pseudocamellia
107	Machilus japonica	140	Pteridium aquilinum	173	Symplocos coreana
108	Maesa japonica	141	Quercus acuta	174	Synurus palmatopinnatifidus
109	Magnolia salicifolia	142	Quercus acutissima	175	Syringa reticulata
110	Magnolia sieboldii	143	Quercus aliena	176	Taxus cuspidata
111	Mallotus japonicus	144	Quercus dentata	177	Thujopsis dolabrata
112	Matteuccia struthiopteris	145	Quercus gilva	178	Tilia japonica
113	Melia azedarach	146	Quercus glauca	179	Trachelospermum asiaticum
114	Meliosma myriantha	147	Quercus myrsinifolia	180	Ulmus davidiana
115	Miscanthus sinensis	148	Quercus salicina	181	Ulmus laciniata
116	Morus australis	149	Quercus serrata	182	Vaccinium hirtum
117	Myrica rubra	150	Quercus variabilis	183	Viburnum dilatatum
118	Nandina domestica	151	Ranunculus acris	184	Viburnum furcatum
119	Neolitsea aciculata	152	Rhododendron nudipes	185	Viburnum odoratissimum
120	Neolitsea sericea	153	Rhododendron wadanum	186	Viburnum phlebotrichum
121	Onoclea orientalis	154	Rubus microphyllus	187	Viburnum wrightii
122	Oreocnide pedunculata	155	Rumex acetosa	188	Vitis coignetiae
123	Osmanthus heterophyllus	156	Salix gracilistyla	189	Weigela hortensis
124	Osmunda japonica	157	Salix sieboldiana	190	Wisteria floribunda
125	Ostrya japonica	158	Salix udensis	191	Zelkova serrata
126	Parasenecio hastatus	159	Sambucus racemosa		
127	Parthenocissus tricuspidata	160	Sasa kurilensis		
128	Petasites japonicus	161	Sasa nipponica		
129	Phellodendron amurense	162	Sasa palmata		
130	Picea jezoensis	163	Sasa senanensis		
131	Picea torano	164	Skimmia japonica		
132	Pieris japonica	165	Smilax china		
